# Chronic Exertional Compartment Syndrome in a High School Soccer Player

**DOI:** 10.1155/2015/965257

**Published:** 2015-07-01

**Authors:** James J. Bresnahan, William L. Hennrikus

**Affiliations:** Penn State Hershey College of Medicine, 500 University Drive, Hershey, PA 17033, USA

## Abstract

Chronic exertional compartment syndrome (CECS) is a relatively rare condition that affects young adult athletes and often causes them to present to the emergency department. If left untreated, those who continue to compete at high levels may experience debilitating leg pain. Physicians may have difficulty differentiating CECS from other syndromes of the lower leg such as medial tibial stress syndrome, stress fractures, and popliteal artery entrapment. The gold standard for diagnosing CECS is intramuscular compartment pressure monitoring before and/or after 10 minutes of exercise. Some patients may choose to stop participation in sports in order to relieve their pain, which otherwise does not respond well to nonoperative treatments. In patients who wish to continue to participate in sports and live an active life, fasciotomy provides relief in 80% or more. The typical athlete can return to training in about 8 weeks. This is a case of a high school soccer player who stopped competing due to chronic exertional compartment syndrome. She had a fascial hernia, resting intramuscular pressure of 30 mmHg, and postexercise intramuscular pressure of 99 mmHg. Following fasciotomy she experienced considerable life improvement and is once again training and playing soccer without symptoms.

## 1. Case

An otherwise healthy 15-year-old female soccer player presented to the emergency department with the complaint of bilateral anterolateral leg pain during exercise that has increased in intensity over the past year. Pain typically developed within minutes and remained for long periods of time, sometimes hours, after exercise. The patient stated that the pain has become increasingly worse even with the moderate exertion of walking or climbing steps at school and was consistently worse in the right leg. She experienced numbness previously during exercise as well as zingers radiating through the anterior and lateral compartments, again more noticeable in her right leg. She has been limited in recreational exercise and has stopped playing high school soccer due to increased discomfort. Activity modification and stretching failed to relieve pain.

*(1) Clinical Examination*. Upon examination, the patient was 158.75 cm (62.5 in) tall and 72.6 kg (160.1 lbs) and showed stable vital signs. There was no tenderness along any of the four leg compartments; however, she exhibited woody compartments. Upon palpation there was a noticeable fascial defect present in the right anterior leg but not the left. The patient was able to walk with a normal gait, as well as heel and toe walk. She was also able to jump evenly with a full range of motion without pain. She had no signs of knee or ankle instability. Forward bend test was normal, her spine showed no curvature, and her upper extremities were supple.

Bilateral X-rays of ankles and feet revealed no evidence of fracture or dislocation. Bone mineralization was normal and there was no ankle joint effusion. The ankle mortise was intact and there was no evidence of focal soft tissue swelling. She exhibited strong bilateral pedal pulses both during and after exercise.

She was referred to the pediatric orthopedic clinic. Lack of improvement with exercise modification along with prolonged pain after exercise led us to consider chronic exertional compartment syndrome (CECS) in addition to medial tibial stress syndrome (MTSS), stress fracture, and popliteal artery entrapment syndrome (PAES) (Table 1). Strong pedal pulses with no calf tenderness during or after running allowed us to rule out PAES without the use of ultrasound. Lack of resting pain or evidence of fracture on X-ray ruled out a stress fracture. To further differentiate CECS and MTSS, we performed pressure measurement in clinic using ethylene chloride (Gebauer Company, Cleveland, OH) as a local anesthetic. We used a Stryker Pressure Monitor System wick catheter (Stryker, Kalamazoo, MI) in the anterior compartment after 10 minutes of jogging. Postexercise examination revealed both anterior leg compartments as woody to touch and firmer than the superficial posterior leg compartments. The palpable fascial defect was even more prominent after exercise. Anterior compartment pressure in the right leg was measured to be 99 mmHg (Table 2, Figure 2), considerably higher than diagnostic indication for fasciotomy which is ≥15 mmHg resting, ≥30 mmHg 1 minute after exercise, or ≥20 mmHg 5 minutes after exercise [1]. 

*(2) Surgical Technique*. Prior to operation while the patient was under general anesthesia, a resting anterior compartment pressure measure was taken on the right leg, which indicated a resting pressure of 29 mmHg (Table 1, Figure 3). This is well outside the normal range for resting compartmental pressure [1]. A 2.5-inch incision was made over the fascial defect on the right anterolateral leg after the leg was elevated and exsanguinated and the tourniquet was set. Blunt dissection allowed access to the fascia and the intermuscular septum was identified as well as the fascial hernia (Figure 4).

The superficial peroneal nerve was identified as it entered the 2 cm fascial defect (Figure 5). The nerve was gently dissected distally and proximally until we found normal nerve. The fascial defect was gently opened distally and proximally until there was a release of the lateral compartment to the fibula tip distally and about 3 cm to the upper fibula proximally (Figure 6). An arthroscopic instrument was used to visualize the fascia release under the skin.

The subcutaneous tissues were gently dissected anteriorly until the anterior compartment was identified (Figure 7). The anterior fascia was opened and then a release of the anterior compartment was done distally to the top of the ankle and proximally to within 3 cm of the knee joint.

The superficial peroneal nerve was allowed to fall back into fatty tissue. The subcutaneous tissues were closed with vicryl. The fascia was left open on the anterior and lateral compartments and her compartmental pressure was remeasured and noted to be normal at 6 mmHg (Figure 8). The skin was closed with monocryl subcuticular running stitch and a sterile compressive dressing was applied.

The patient was advised to bear weight as tolerated and to use crutches as needed in the immediate postoperative period. We followed up with our patient at 5 days, 4 weeks, and 3 months. At 5 days following operation, the surgical site was healing well with no signs of infection. She regained full range of motion and appeared neurovascularly intact. At 4 weeks she continued to improve without complications. The surgical site was healing well and without bruising or swelling. Our patient regained strength and self-reported a noticeable improvement in her ability to walk and climb stairs compared to preoperation. The patient was allowed to walk, swim, and ride a bike without great exertion. She was allowed to run once again at 8 weeks' postoperative time. At 3 months following operation, our patient was noticeably better with greatly improved exercise capacity. She has returned to recreational running and playing high school soccer.

## 2. Discussion

The emergency room physician is often the first clinician to diagnose compartment syndrome, which can be classified as acute or chronic. Acute compartment syndrome is attributed to various causes (i.e., fracture, ischemia, improper casting, etc.) and often presents with extreme pain in a passive state as an emergency. Fasciotomy is the gold standard for relieving symptoms associated with acute compartment syndrome [2].

In contrast, CECS is less emergent but highly disruptive for people with an active lifestyle. CECS usually occurs in the lower leg and most likely results from increased pressure in one or more muscle compartments. CECS may result in neurovascular abnormalities causing extreme pain during and after exercise and often causes athletes to stop competing and exercising. The prevalence of CECS is unknown and it is likely that many people who suffer from CECS modify their activities in a way to reduce pain without ever seeking medical intervention. It is also possible that some people with CECS are not taken seriously by athletic trainers, coaches, and primary care physicians to whom symptoms are first presented [3].

**Figure 1:**
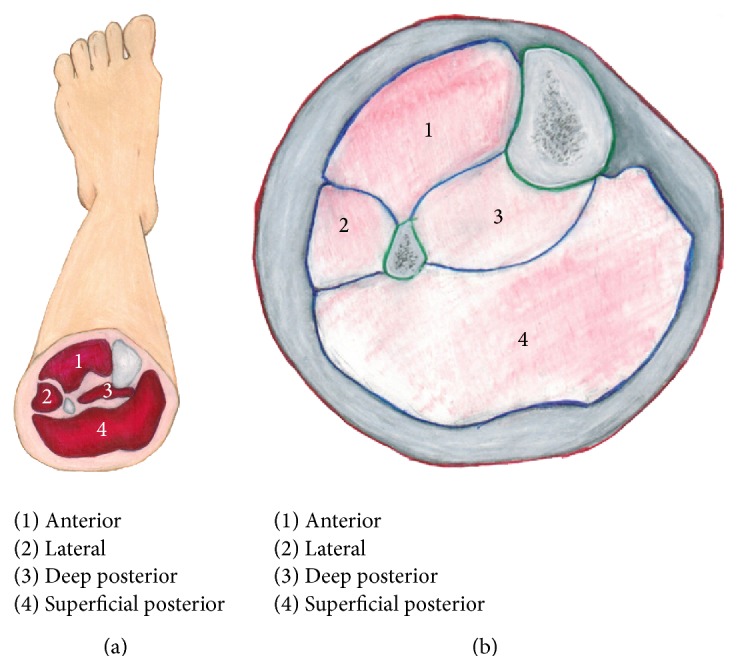


**Figure 2:**
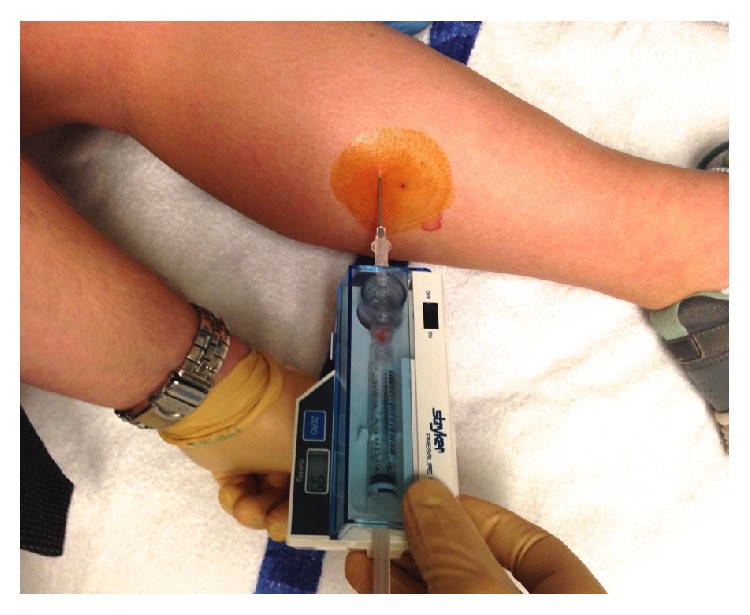


**Figure 3:**
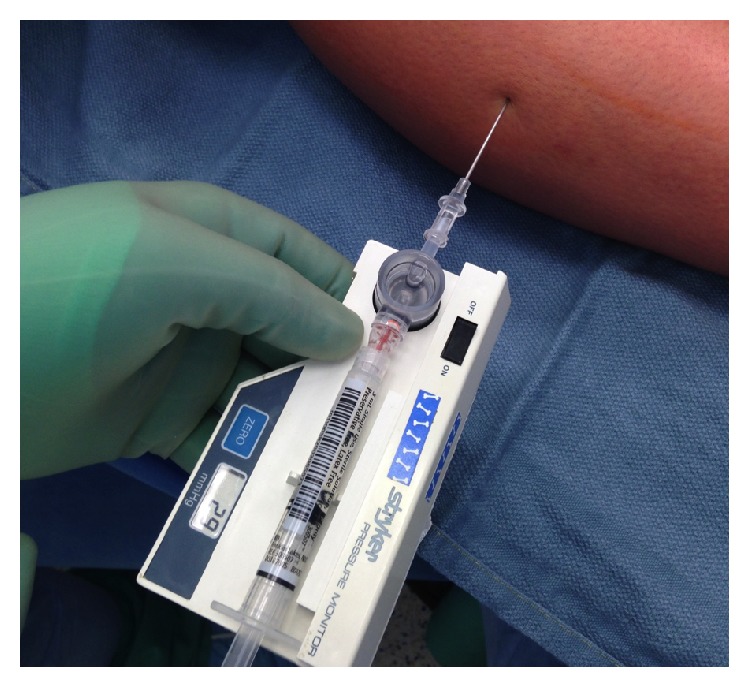


**Figure 4:**
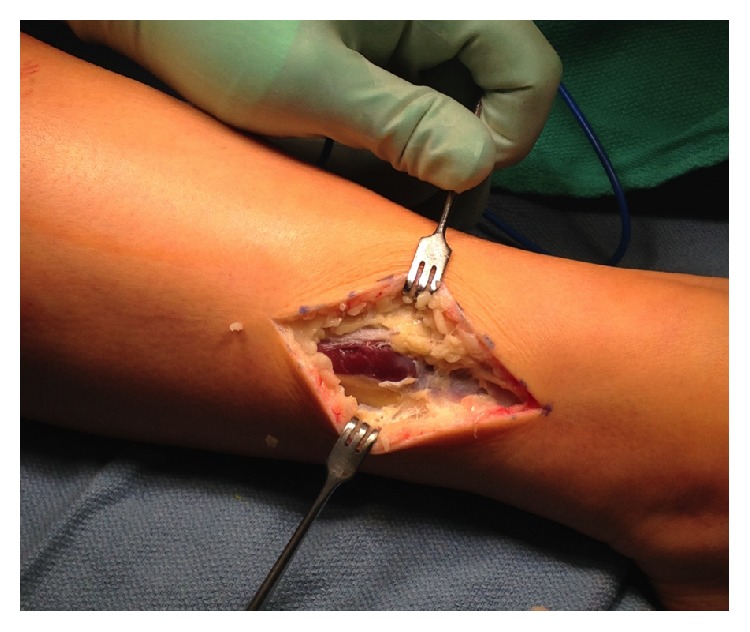


**Figure 5:**
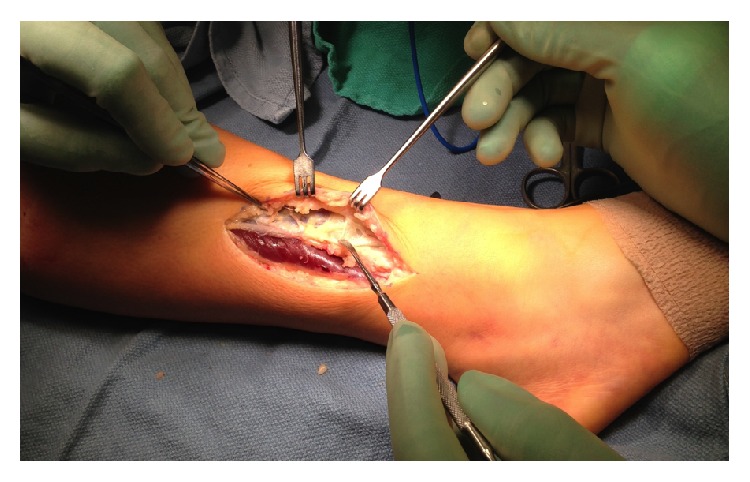


**Figure 6:**
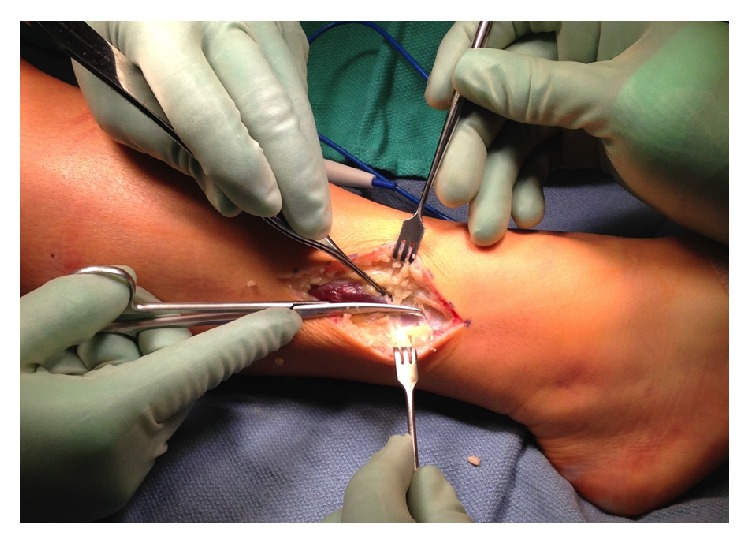


**Figure 7:**
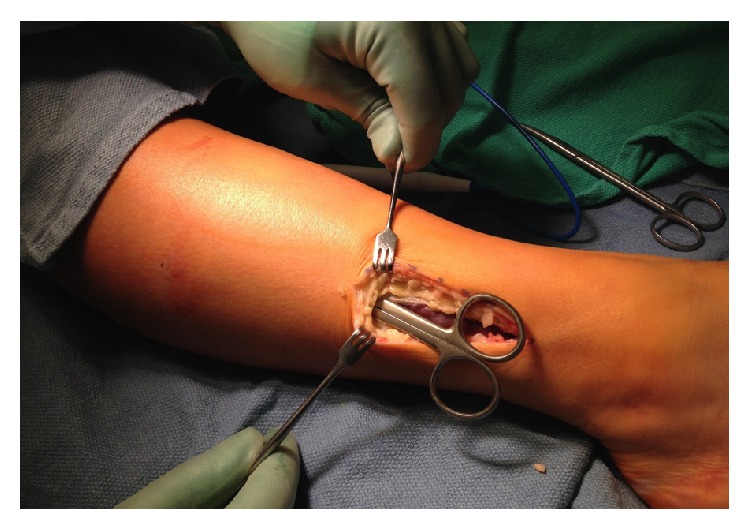


**Figure 8:**
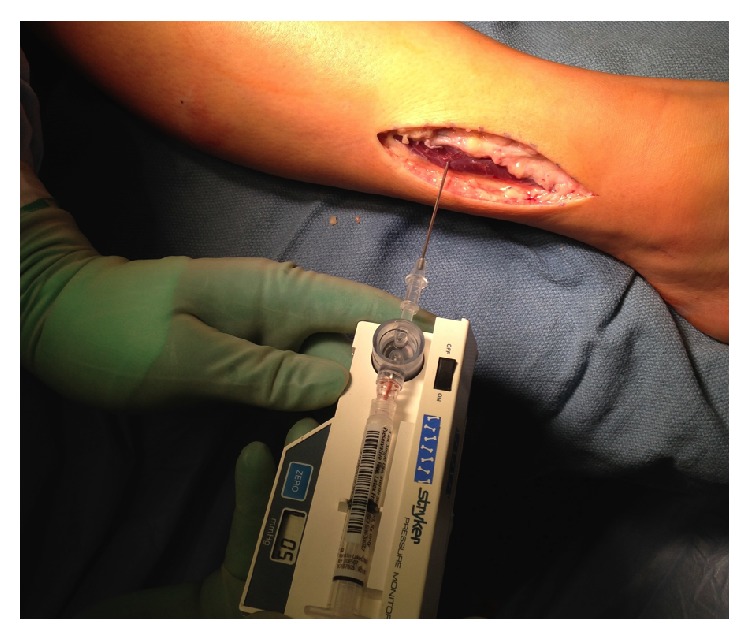


CECS most often affects the lower leg, which is divided into four compartments: anterior, lateral, deep posterior, and superficial posterior (Figure 1). Of these four compartments, the anterior and lateral compartments are most commonly affected [4].

### 2.1. Pathophysiology

The pathophysiology is not completely understood. It has been previously noted that CECS patients have decreased blood flow and oxygenation in their legs [5]. It is thought that as blood flow increases to the leg muscles during exercise, they expand more than the fascia passively allows. Muscle fibers have been shown to swell up to 20 times their resting size during strenuous repetitive activities [6]. The increased pressure makes perfusion more difficult and the body is unable to meet the metabolic demands of the muscles. This increased pressure causes pain until the compartment returns to normal and blood flow is restored.

### 2.2. Clinical Presentation

CECS typically presents in young adult athletes, often those who run considerable amounts. The pain is usually described as being specific to a location that corresponds with a muscle region, usually in the lower leg. Pain begins within a few minutes of starting a strenuous activity; patients are often able to tell an exact time or mileage if they are vigilant about tracking their exercise. The pain is generally described as aching, squeezing, cramping, or tightness. Pain typically subsides, but not immediately after stopping the insulting activity. It often takes 10–20 minutes and pain may linger for hours [7].

CECS can be a bilateral condition. Although the literature varies as to the percentage of people affected bilaterally, Detmer et al. suggest that patients may not realize they suffer from bilateral CECS if one side is worse than another [8]. In the current case, the patient's symptoms were predominantly in the right leg. The patient will be followed up for potential CECS in the left leg.

Muscle hernias are also commonly present in 20–60% of cases [1, 9–11]. However, hernias are also seen in other causes of lower leg pain, decreasing their diagnostic potential [1]. Leg muscles may appear to be bulky and firm in appearance [11]. Abnormal distal pulses are not common and should lead one to consider alternate diagnoses such as PAES [8, 11].

### 2.3. Treatment

Diagnostic criteria for CECS include one or more of the following intramuscular pressure readings: ≥15 mmHg at rest; ≥30 mmHg 1 minute after exercise; ≥20 mmHg 5 minutes after exercise [1].

Nonoperative interventions such as physical therapy, stretching, rest, and anti-inflammatory medication often fail to resolve or prevent pain. Fasciotomy, although invasive, has been successfully used in the treatment of CECS since it was first performed in 1956 [10]. Surgical results are better in CECS cases involving the lateral and anterior compartments when compared to posterior compartments [12, 13]. Fasciotomy is effective in the treatment of CECS with a success rate of 80% or greater [3, 8, 14, 15]. One study looking at a population of military men under extreme physical demand showed persistence of symptoms to some degree in up to 50% of patients. This study, also found that surgical revision may be needed in up to 6% of patients [15]. This study highlighted the high physical demand and increased body weight due to carrying equipment, yet patients experienced an 80% successful return to activity. We believe this study further shows the success of fasciotomy in CECS albeit slightly less positive than literature related strictly to young athletes. Athletes are able to return to a similar level of activity they were able to do before surgery at 10.6 weeks on average but range from 6 to 12 weeks depending on severity and number of compartments released. It is generally regarded as a decision to be made between the orthopedic surgeon and the patient, but on average patients return to full levels of activity at 8.25 weeks [16]. In the current case the patient returned to normal activity 12 weeks following operation.

## 3. Conclusion

CECS is a relatively rare, although possibly underdiagnosed, condition that most commonly presents in young adult athletes. The most likely cause of CECS is the expansion of muscle tissue during exercise resulting in the buildup of metabolites and nerve entrapment. Those affected may experience severe and debilitating pain that often starts at a specific time during exercise and does not subside during or after exercise. The diagnosis can be suggested by history of present illness, physical examination. Radiographic studies (X-ray, MRI, etc.) are used to rule out other diagnoses such as stress fracture. The diagnostic test of choice is compartment pressure measures before and after exercise. Fasciotomy is the treatment of choice and allows most athletes to return to competition in approximately 8 weeks. CECS should be considered by emergency medicine physicians, coaches, trainers, therapists, and others for patients with more serious pain associated with exercise that does not cease with rest.

## Figures and Tables

**Table 1:** Differential diagnosis for lower leg pain.

	CECS	MTSS	PAES	Stress fracture
Clinical picture	Pain after a certain period of activity, prolonged pain after rest, sometimes with palpable fascial hernia.	Pain that ceases with rest, typically on the inner leg.	Constant pain, decreased pedal pulses, signs of cyanosis.	Localized tenderness

Diagnosis	Intramuscular compartment pressure measurement	Clinical	Ultrasound	X-ray

Treatment	Fasciotomy	Rest, ice, elevation	Surgical release of tendons	Rest, ice, elevation

CECS: chronic exertional compartment syndrome; MTSS: medial tibial stress syndrome; PAES: popliteal artery entrapment syndrome.

**Table 2:** Intramuscular pressure readings at rest and exercise in different compartments of the right leg.

Compartment	Pressure at rest (mmHg)	Pressure after exercise (5 min) (mmHg)
Anterior	29	99
